# A case of pericardial schistosomiasis and non-Hodgkin high grade B-cell lymphoma

**DOI:** 10.4102/sajid.v38i1.524

**Published:** 2023-09-29

**Authors:** Michael J. Boyd, Marc Mendelson, Sipho K. Dlamini, Sean Wasserman, Ghaalied Fakier, Riyaadh Roberts, Nectarios S. Papavarnavas

**Affiliations:** 1 Faculty of Medicine and Health Sciences, Stellenbosch University, Cape Town, South Africa; 2 Division of Infectious Diseases & HIV Medicine, Department of Medicine, Faculty of Health Sciences, University of Cape Town, Cape Town, South Africa; 3 Department of Medicine, Faculty of Health Sciences, University of Cape Town, Cape Town, South Africa; 4 Wellcome Centre for Infectious Diseases Research in Africa, Faculty of Health Sciences, University of Cape Town, Cape Town, South Africa; 5 Division of Anatomical Pathology, Department of Pathology and National Health Laboratory Services, Faculty of Health Sciences, University of Cape Town, Cape Town, South Africa

**Keywords:** schistosomiasis, pericardial schistosomiasis, *Schistosoma haematobium*, non-Hodgkin lymphoma, pericardial effusion

## Abstract

Chronic schistosomiasis affects either the genitourinary or gastrointestinal tract. Rarely, schistosomes cause ectopic disease, such as in the case of a South African woman from a non-endemic province, who presented with suspected pericardial tamponade because of tuberculosis. However, histology and polymerase chain reaction from pericardial biopsy confirmed *Schistosoma haematobium.* A finding of mediastinal non-Hodgkin lymphoma came to light when our patient’s clinical condition unexpectedly deteriorated.

**Contribution:**

This case highlights an unusual manifestation of schistosomiasis.

## Introduction

*Schistosoma* species infect approximately 230 million people worldwide, causing an estimated 280 000 deaths per annum.^1^ Infection by *Schistosoma* trematodes can produce both acute and chronic manifestations. Skin penetration by schistosomal cercariae may cause a cercarial dermatitis (swimmer’s itch) at the time of infection, which can be followed 3–6 weeks later by acute schistosomiasis, also known as Katayama fever, a transient hypersensitivity reaction to migrating schistosomulae.^2^ A sustained inflammatory, granulomatous, and fibrotic reaction directed towards trapped schistosome ova in the genitourinary (*Schistosoma haematobium, Schistosoma intercalatum*) or gastrointestinal (*Schistosoma mansoni, Schistosoma japonicum*) tracts, causes chronic manifestations.^3,4,5^ Ectopic schistosomiasis is rare and presents a diagnostic challenge to clinicians, especially when patients present in a non-endemic area and a lack of attention is paid to eliciting a detailed travel history. Schistosomiasis was first noticed in the Eastern Cape province of South Africa in 1863, and is endemic in the northern and eastern parts of South Africa, with the most common species being *S. haematobium*.^6,7,8^ It is estimated that approximately 4 million people in South Africa are at risk of infection.^6^ We report an unusual case of pericardial schistosomiasis in a non-endemic area of South Africa.

## Case report

**FIGURE 1:**
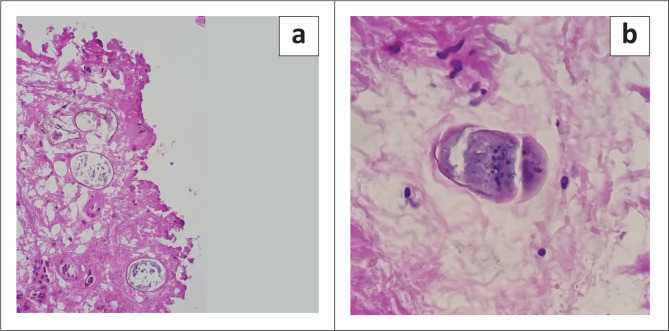
*Schistosoma* ova: (a) Non-viable ova, 400 × magnification, (b) viable ova, 400 × magnification.

A previously well, HIV-negative, 44-year-old woman presented to her primary care facility in the Western Cape province of South Africa with a 3-month history of shortness of breath and dry cough. She denied weight loss, fever, or night sweats. An X-ray illustrated a left-sided pleural effusion and she was empirically started on tuberculosis treatment based on a monocyte predominant pleural fluid with a high adenosine deaminase of 56.2 U/L. One week later she presented with cardiac tamponade and was urgently referred to a tertiary hospital for further management. A pericardial window was performed and 200 mL of fluid was aspirated. Tuberculosis culture and Xpert^®^ MTB/RIF Ultra of the pleural fluid, as well as the pericardial tissue were negative. Pericardial fluid cytological examination did not reveal malignant cells. Histology of pericardial tissue showed inflammation and poorly formed granulomas surrounding suspected *Schistosoma haematobium* ova as did a Ziehl-Neelsen stain, there were no signs of lymphoma (Figure 1).

On further history, it was revealed that she had travelled to Israel 4 years prior to presentation, swimming in the river Jordan, a non-endemic area for schistosomiasis. However, her childhood was spent swimming in rivers and lakes in the Eastern Cape province of South Africa, a highly endemic area for schistosomiasis.

An immunofluorescence assay for *Schistosoma* cercariae was performed as our laboratory does not offer ova immunofluorescent assay antigen testing. Cercarial antibodies were negative on two separate occasions as were repeated stool and urine microscopy for ova, taken between 10:00 and 14:00. A *Schistosoma* genus polymerase chain reaction (PCR) of the pericardial tissue was positive for *Schistosoma* species, but could not differentiate between *S. haematobium* and *S. mansoni*.

The patient’s symptoms improved following her procedure. She was given 3 days of praziquantel at 20 mg/kg twice daily for 3 days. However, on day 27 of her admission, she developed acute cardiorespiratory failure. Urgent echocardiography and computerised thoracic pulmonary arteriography (CTPA) revealed a mediastinal mass causing marked cardiac compression and obstruction of both the superior vena cava and left brachiocephalic vein rather than pericardial fluid re-accumulation or a pulmonary embolus. A computed tomography-guided biopsy of the mediastinal mass revealed a non-Hodgkin high grade B-cell lymphoma on histology. Her tuberculosis treatment was discontinued.

## Discussion

Pericardial involvement is a rare complication of chronic schistosomiasis infection. As access to pericardial biopsy in low- and middle-income countries is limited, it may be under-reported. Infrequently, eggs can find their way outside of primary sites, such as in the case of neuroschistosomiasis or pulmonary schistosomiasis.^9^ Involvement of the myocardium or pericardium in chronic schistosomiasis infection is uncommon.^10,11,12,13,14,15^ Clark and Graef described ova in myocardial tissue surrounded by fibrosis at postmortem.^11^ The first case of pericardial schistosomiasis was reported in a 16-year old from Durban in KwaZulu-Natal, South Africa presenting in heart failure in 1979.^14^ Like our patient, she was treated initially for tuberculosis, but subsequent pericardial histopathology revealed widespread eosinophilic inflammation and granulomatous change surrounding *S. haematobium* ova. A study from Egypt identified 15 patients with *S. mansoni* infection with pure, right-sided endomyocardial fibrosis, from 10 000 consecutive patients who underwent echocardiography at a single hospital over a 3-year period.^15^ Histopathological confirmation of pericardial schistosomiasis was obtained in one of the patients, but all had evidence of marked fibrosis on endomyocardial biopsy.

It remains uncertain how Schistosoma ova find their way into the pericardium. Ova distribution outside of the characteristic genitourinary, gastrointestinal or pre-hepatic sites is rare. In neuroschistosomiasis, migration of ova may be via the portal-mesenteric or pelvic systems through pre-existing pulmonary shunts or through the development of portal hypertension with resultant shunting via the valveless Batson plexus.^16^ This plexus connects the portal venous system to the inferior vena cava that supplies the spinal cord and cerebral veins.^16,17^ Furthermore, patients with established schistosomiasis-induced hepatic fibrosis may have pulmonary involvement as retention of ova within the portal system can elicit a host response leading to portal hypertension with resultant shunts that facilitate their further dissemination.^18^ Van der Horst proposed that pericardial schistosomiasis may occur because of embolisation of ova into the inferior vena cava, or eggs that travel via the lymphatics into the systemic circulation.^14^

The aetiology of the pericardial effusion is undefined but has three main differentials. Firstly, biopsy-proven pericardial schistosomiasis may have induced an inflammatory effusion. Secondly, it may have been because of lymphoma involving the pericardium despite the aspirated fluid and pericardial biopsy not showing lymphomatous changes. Thirdly, lymphoma adjacent to the surrounding tissues may have resulted in inflammation with effusion.

**FIGURE 2:**
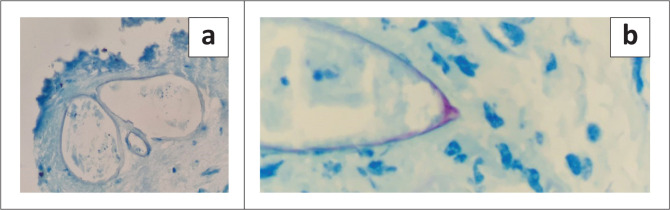
Ziehl-Neelsen Stain: (a) 400 × magnification, (b) > 400 magnification showing acid fast positivity and terminal spine, suggestive of *S. haematobium.*

Serological testing for cercariae with immunofluorescent assay has low sensitivity (48% – 87%) but high specificity (95%).^19^ Serology against ova antigens was not available. The histopathology of pericardial biopsies was consistent with the predominant species found in South Africa, *S. haematobium* infection,^20^ as the ova had a terminal spine. Interestingly, Ziehl-Neelsen histochemical staining was positive, a feature more frequently associated with *S. mansoni* or other Schistosoma species (Figure 2).^21^ This may be because of variation in stain preparation or stain artefact. Other possibilities, thought to be less likely, include *S. intercalatum*, not usually seen in South Africa, which also has a terminal spine; or newly described hybrid Schistosoma species, which may show characteristics of multiple species.^22^

The World Health Organization and other guidelines recommend single dose praziquantel 40 mg/kg to treat schistosomiasis in endemic countries.^23,24,25^ However, there are a number of studies using different dosing strategies.^26^ We opted for a regimen based on evidence supporting 40 mg/kg per day for 3 days because of the severe nature of the disease.^27,28,29,30^ Corticosteroids are used in neuroschistosomiasis to dampen inflammation.^16^ We opted to discontinue corticosteroids, initially given for the suspected tuberculosis pericarditis as to the best of our knowledege, there is no literature on how best to treat patients with pericardial schistosomiasis. However, the patient was later diagnosed with non-Hodgkin’s lymphoma and was then given corticosteroids, which may have also helped to dampen the inflammation from the pericardial schistosomiasis.

The finding of mediastinal non-Hodgkin’s lymphoma in our patient was unanticipated. Squamous cell carcinoma of the bladder following chronic inflammation is a well-described complication of *S. haematobium* infection.^31^ However, a number of case reports suggest a link between schistosomiasis infection and lymphoma.^32,33,34,35,36,37^ One such report, was of a patient with B-cell lymphoma and *S. haematobium* whose lymphoma subsequently regressed after treatment with praziquantel. This response led to the authors proposing a causal link between schistosomiasis and lymphoma.^38^ To our knowledge, no other reports have described this phenomenon. At present, any pathophysiological link between our case of pericardial schistosomiasis with concurrent non-Hodgkin high grade B-cell lymphoma remains speculative.

## Conclusion

This report highlights the rare manifestation of pericardial schistosomiasis, the true incidence of which remains unknown. Although acute schistosomiasis is commonly considered in returning travellers to high-income countries from endemic areas, a history of living in an endemic location is not always considered in a patient presenting in a non-endemic area within the same country. A potential correlation between schistosomiasis and lymphoma requires further study.

## Learning points

- Ectopic migration of *Schistosoma* ova is a rare manifestation of a common infection.
- A proper travel history including within-country travel and risk exposures is a critical part of the diagnostic evaluation.
- Serological assays directed at one antigen in the *Schistosoma* life cycle do not rule out chronic schistosomiasis, particularly if that antigen is cercarial.
- Any link between lymphoma and schistosomiasis remains undefined.
